# A numerical approach for a discrete Markov model for progressing drug resistance of cancer

**DOI:** 10.1371/journal.pcbi.1006770

**Published:** 2019-02-19

**Authors:** Masayuki Maeda, Hideaki Yamashita

**Affiliations:** Graduate School of Social Sciences, Tokyo Metropolitan University, Tokyo, Japan; University of California Irvine, UNITED STATES

## Abstract

The presence of treatment-resistant cells is an important factor that limits the efficacy of cancer therapy, and the prospect of resistance is considered the major cause of the treatment strategy. Several recent studies have employed mathematical models to elucidate the dynamics of generating resistant cancer cells and attempted to predict the probability of emerging resistant cells. The purpose of this paper is to present numerical approach to compute the number of resistant cells and the emerging probability of resistance. Stochastic model was designed and developed a method to approximately but efficiently compute the number of resistant cells and the probability of resistance. To model the progression of cancer, a discrete-state, two-dimensional Markov process whose states are the total number of cells and the number of resistant cells was employed. Then exact analysis and approximate aggregation approaches were proposed to calculate the number of resistant cells and the probability of resistance when the cell population reaches detection size. To confirm the accuracy of computed results of approximation, relative errors between exact analysis and approximation were computed. The numerical values of our approximation method were very close to those of exact analysis calculated in the range of small detection size M = 500, 100, and 1500. Then computer simulation was performed to confirm the accuracy of computed results of approximation when the detection size was M = 10000,30000,50000,100000 and 1000000. All the numerical results of approximation fell between the upper level and the lower level of 95% confidential intervals and our method took less time to compute over a broad range of cell size. The effects of parameter change on emerging probabilities of resistance were also investigated by computed values using approximation method. The results showed that the number of divisions until the cell population reached the detection size is important for emerging the probability of resistance. The next step of numerical approach is to compute the emerging probabilities of resistance under drug administration and with multiple mutation. Another effective approximation would be necessary for the analysis of the latter case.

## Author summary

Drug therapies for cancer have dramatically succeeded since molecular-targeted drugs have been introduced in medical practice; however, drug treatment often fails owing to the emergence of drug-resistant cells. A variety of approaches, including mathematical modeling, has been undertaken to clarify the mechanism of resistance and subsequently avoid resistance to therapy. This paper proposes one of the mathematical approaches that uses a stochastic model and provides the emerging probabilities of resistance at detection size.

## Introduction

Oncogenic pathways have been investigated using molecular biology techniques, which have helped elucidate the molecular mechanism of cancer growth, invasion, and metastasis among other aspects. The findings from these investigations have encouraged the development of anti-cancer drugs that inhibit specific oncogenic pathway and have helped improve clinical outcomes dramatically [1] [2]. But there exists some percentage of patients who have no response to these kinds of drugs. One of the reasons for the lack of response is the point mutation of a specific gene. Cancer cells mutate and acquire resistance to the anti-cancer drug, posing a significant obstacle for curing cancer [3] [4].

Several recent studies have attempted to understand the proliferation of cancer cells by employing mathematical models for the process of biological evolution [4] [5]. The mutation occurs randomly in the cell population and the number of resistant cells in the population increases as the cancer cells grow. Because expanding process of resistant cells in cancer cell population is like the dynamics of biological evolution, this process of expanding mutation cells can be viewed as an evolutionary process within the body occurring within a short span of time [5]. Mathematical models are often used to elucidate the dynamics of evolutionary process and have been studied to understand the mechanism through which cancer cells develop drug resistance [4] [6] [7] [8] [9] [10].

Iwasa et al. [11] analyzed the dynamics of resistant mutants in the exponential growth of cancer cells. The authors used a continuous-time branching process to calculate the probability of resistance, and the probability distribution of resistant cells when the population of cancer cells attains a certain detection size in the absence of therapy. They observed that the probability of resistance is an increasing function with the product of detection size and mutation rate. They concluded that the probability of resistance and the average number of resistant cells increase with the number of cell divisions over the course of the cancer.

Haeno et al. [12] extended Iwasa’s model to cancer cells carrying two mutations. They also used a continuous-time branching process to calculate the probability of formation of at least one cell carrying two mutations, and the probability distribution when the population reaches a certain size. Their findings were similar to those from Iwasa’s study.

Foo et al. [13] [14] modelled the cancer cell population during treatment with a continuous-time birth and death process. They measured the effect of drugs in reducing the proliferation rate of drug-sensitive cells. They studied resistance dynamics during therapy under a general time-varying treatment schedule. They coupled their stochastic framework with pharmacokinetic models incorporating the processes of drug absorption and elimination within the body. They calculated the probability of resistance arising during continuous and pulsed administration strategies. They used their estimates of probability of resistance and population size of drug-resistant cells to determine an optimum drug administration schedule that would minimize the risk of resistance.

The mathematical approach used in these models were analytical ones. The probability of resistance was obtained by solving differential equations and confirmed by computer simulations. To obtain the analytical solution, various ways to derive the solution of equations were devised. For example, the authors in [11] [12] [13] [14] handle the cell size as continuous variables in their calculations, though it is considered appropriate for addressing the discrete state space when the dynamics of cell size is discussed.

The purpose of this paper is to present numerical approach for computing the emerging probability of resistant cells. We model the process of cancer progression through a discrete state space, continuous time Markov chain. Then, we transform it into an embedded Markov chain to reduce the computational effort for computing the emerging probabilities of resistant cells.

This paper first explains our models for computing the number of resistant cells. Then the two ways of computation are presented: Exact analysis and aggregation approximation. The approximation method is introduced as efficient way to compute. Second, the computed values of exact analysis and those of approximate are compared. At the same time the computed values of exact analysis also are compared with those of previous study. The relative errors are used for evaluating the accuracy of these computed values. Third, values of computer simulation are compared with those of approximation. This comparison is performed at detection size over 10000. The 95% confident intervals are used to confirm and evaluate the accuracy of computed values of approximation. The execution time of both methods are also compared. Lastly, the parameter dependency on the computed values of emerging probabilities of resistant cells. The parameter dependency of division rate, death rate and mutation rate are investigated by computing the probabilities of resistance with changing these values of parameters. Factors of effecting the emerging probabilities of resistance are discussed.

## Materials and methods

### Cancer progression model

Consider an expanding cancer cell population. There are two types of cancer cells: drug-sensitive and drug-resistant. The sensitive cells divide and die at a rate of λ and *μ*, respectively. The probability of mutation when a sensitive cell divides (i.e., the probability of formation of a resistant cell) is *γ*, and the probability of formation of a sensitive cell is 1 − *γ*. The resistant cells divide and die at a rate of *α* and *β*, respectively. Our objective is to obtain the distribution of resistant cells when the total cell population reaches detection size, which is denoted by *M*. Let us denote the total number of cells (both sensitive and resistant) and the number of resistant cells by *m* and *n*, respectively. Then, the number of sensitive cells increases at the rate of λ(1 − *γ*)(*m* − *n*) and decreases at the rate of *μ*(*m* − *n*). The number of resistant cells increases at the rate of λ*γ*(*m* − *n*) + *αn* and decreases at the rate of *β* *n*. Let us consider the two-dimensional Markov chain with states (*m*, *n*), where *m* = 0, 1, 2, ⋯, *M*; *n* = 0, 1, 2, ⋯, *m*. Let us consider the process starting at the state (*m*, *n*) = (1, 0). The process can end either at extinction (*m* = 0) or when the cell population reaches detection size (*m* = *M*).

Let us denote the state of the process (*m*, *n*) after the *t*-th event (cell division or death) as (*m*_*t*_, *n*_*t*_). The transition probabilities of this process are given as follows:

$$P_{r}\{(m_{t+1},n_{t+1})=(i+1,j)|(m_{t},n_{t})=(i,j)\}=\lambda (1-\gamma )(i-j)/\Gamma_{i,j}$$ (1)

$$P_{r}\{(m_{t+1},n_{t+1})=(i+1,j+1)|(m_{t},n_{t})=(i,j)\}=\{\lambda \gamma (i-j)+j\alpha \}/\Gamma_{i,j}$$ (2)

$$P_{r}\{(m_{t+1},n_{t+1})=(i-1,j)|(m_{t},n_{t})=(i,j)\}=\mu (i-j)/\Gamma_{i,j}$$ (3)

$$P_{r}\{(m_{t+1},n_{t+1})=(i-1,j-1)|(m_{t},n_{t})=(i,j)\}=j\beta /\Gamma_{i,j}$$ (4)

Here *Γ*_*i*,*j*_ = (*i* − *j*)(λ + *μ*) + *j*(*α* + *β*) is the sum of the rates, normalizing the total probability to 1. Note that the Markov chain (*m*, *n*) is homogeneous.

### Transition probability matrix

Let us define the set of states in which the total number of cells is *i*, {(*i*, 0),(*i*, 1) ⋯ (i, i)} as level *i*. Let us denote the transition probability sub-matrix from level *i* to level *i* + 1, and from level *i* to level *i* − 1 as *P*_*i*_ and *Q*_*i*_, respectively. The element of *P*_*i*_ in the *k*-th row and *i*-th column is the transition probability from (*i*, *k*) to (*i* + 1, *k*). The corresponding element of *Q*_*i*_ is the transition probability from (*i*, *k*) to (*i* − 1, *k*).

*P*_*i*_ and *Q*_*i*_ are expressed as follows:

$$\begin{array}{cc} P_{i}= & \left(\begin{array}{cccccccc} \frac{i\lambda (1-\gamma )}{\Gamma_{i0}} & \frac{i\lambda \gamma }{\Gamma_{i0}} & 0 & \cdots & \cdots & \cdots & \cdots & 0\\ 0 & \frac{\left(i-1)\lambda (1-\gamma \right)}{\Gamma_{i1}} & \frac{(i-1)\lambda \gamma +\alpha }{\Gamma_{i1}} & 0 & \cdots & \cdots & \cdots & 0\\ 0 & 0 & \frac{\left(i-2)\lambda (1-\gamma \right)}{\Gamma_{i2}} & \frac{(i-2)\lambda \gamma +2\alpha }{\Gamma_{i2}} & \cdots & \cdots & \cdots & 0\\ \cdots \cdots \cdots \cdots \cdots \cdots \cdots \cdots \cdots \cdots \cdots \cdots \cdots \\ 0 & 0 & \cdots & \cdots & \cdots & \frac{\lambda (1-\gamma )}{\Gamma_{ii-1}} & \frac{\lambda \gamma +(i-1)\alpha }{\Gamma_{ii-1}} & 0\\ 0 & 0 & \cdots \cdots \cdots \cdots \cdots \cdots & 0 & \frac{i\alpha }{\Gamma_{ii}} \end{array}\right) \end{array}$$

$$\begin{array}{cc} Q_{i}= & \left(\begin{array}{cccccc} \frac{i\mu }{\Gamma_{i0}} & 0 & 0 & \cdots & \cdots & 0\\ \frac{\beta }{\Gamma_{i1}} & \frac{(i-1)\mu }{\Gamma_{i1}} & 0 & \cdots & \cdots & 0\\ 0 & \frac{2\beta }{\Gamma_{i2}} & \frac{(i-2)\mu }{\Gamma_{i2}} & \cdots & \cdots & 0\\ \cdots \cdots \cdots \cdots \cdots \cdots \cdots \\ 0 & 0 & 0 & \cdots & \frac{(i-1)\beta }{\Gamma_{ii-1}} & \frac{\mu }{\Gamma_{ii-1}}\\ 0 & 0 & \cdots \cdots & 0 & \frac{i\beta }{\Gamma_{ii}} \end{array}\right) \end{array}$$

where *P*_*i*_ is the (*i* + 1) × (*i* + 2) matrix, and *Q*_*i*_ is the (*i* + 1) × *i* matrix. We can now express the transition probability matrix of the process (*m*, *n*) by using *P*_*i*_ and *Q*_*i*_ as follows:

$$\begin{array}{c} S=(\begin{array}{ccccccccc} 1 & 0 & 0 & 0 & 0 & \cdots & \cdots & \cdots & 0\\ Q_{1} & 0 & P_{1} & 0 & 0 & \cdots & \cdots & \cdots & 0\\ 0 & Q_{2} & 0 & P_{2} & 0 & \cdots & \cdots & \cdots & 0\\ 0 & 0 & Q_{3} & 0 & P_{3} & \cdots & \cdots & \cdots & 0\\ \cdots \cdots \cdots \cdots \cdots \cdots \cdots \cdots \\ 0 & \cdots & \cdots & \cdots & \cdots & \cdots & Q_{M-1} & 0 & P_{M-1}\\ 0 & \cdots & \cdots & \cdots & \cdots & \cdots & \cdots & 0 & I_{M} \end{array}) \end{array}$$

Note that the states in levels 0 and *M* are absorbing states.

### Exact analysis

We take the submatrix *T* from *S* as follows:

$$\begin{array}{c} T=(\begin{array}{cccccccc} 0 & P_{1} & 0 & 0 & \cdots & \cdots & \cdots & 0\\ Q_{2} & 0 & P_{2} & 0 & \cdots & \cdots & \cdots & 0\\ 0 & Q_{3} & 0 & P_{3} & \cdots & \cdots & \cdots & 0\\ \cdots \cdots \cdots \cdots \cdots \cdots \\ 0 & \cdots & \cdots & \cdots & \cdots & \cdots & Q_{M-1} & 0 \end{array}) \end{array}$$

The submatrix corresponds to the transition probability matrix from transient states to transient states.

Then, *m*_*t*_ denotes the total number of cancer cells and *n*_*t*_ denotes the number of resistant cells after the *t*-th event (cell division or death). Here, we define (*m*_*t*_, *n*_*t*_) as the state of the number of total cells and resistant cells after the *t*-th event. In addition, the probability being at the state is expressed as $\pi_{\left(i,j\right)}^{t}=Pr\{(m_{t},n_{t})=(i,j)\}$.

Now, we define the probability distribution vector:

$$\begin{array}{c} \pi_{i}^{t}=\left({\pi^{t}}_{\left(i,0\right)}{,\pi^{t}}_{\left(i,1\right)},{\pi^{t}}_{\left(i,2\right)},\cdots ,{\pi^{t}}_{\left(i,i-1\right)},{\pi^{t}}_{\left(i,i\right)}\right) \end{array}$$

Here, we consider the process starting at one sensitive cell and no resistant cell, so the initial state of the process is (*m*_0_, *n*_0_) = (1, 0) i.e.,

$$\begin{array}{c} \{\begin{array}{ll} \pi^{0}{}_{i}=\left(1,0\right) & \left(i=1\right)\\ \pi^{0}{}_{i}=0 & \left(i\neq 1\right) \end{array} \end{array}$$

Our objective is to obtain the probability distribution and the emerging probabilities of resistant cells when the total number of cells reaches *M*. As level 0 and level *M* are absorbing states, the emerging probability of resistant cells is expressed by $\underset{t\to \infty }{\text{lim}}\frac{\pi_{\left(M,j\right)}^{t}}{1-\pi_{\left(0,0\right)}^{t}}$ (*j* = 0, 1, 2, 3, ⋯, *M*).

In general, to calculate the distribution of probability in the absorbing states, we should obtain the fundamental matrix as shown below:

$$\begin{array}{c} I+T+T^{2}+T^{3}+\cdots ={\left(I-T\right)}^{-1} \end{array}$$ (5)

The matrix *T* is large, and the calculation of the fundamental matrix involves high computational complexity as the matrix becomes large. Therefore, we propose another algorithm to reduce complexity.

Let ***π***_*i*_ = (*π*_(*i*,0)_, *π*_(*i*,1)_, *π*_(*i*,2)_, ⋯, *π*_(*i*,*i*−1)_, *π*_(*i*,*i*)_) be the probability distribution of the process at first arrival to level *i*, where $\sum_{k=0}^{i}\pi_{(i,k)=1}$. The discrete-time chain {***π***_*i*_} is said to be embedded in {$\pi_{i}^{t}$}, so it is referred to as embedded Markov chain. Then the probability distribution when the total number of cells reaches *M* is equivalent to ***π***_*M*_. Let us now denote the probability matrix that the state at first arrival to level *i* + 1 is (*i* + 1, ⋅) under the condition that the state at first arrival to level *i* is (*i*, ⋅) by *F*_*i*_. There are two paths of transition for the state in level i to reach level i+1. In one, the cells transition directly from level *i* to level *i* + 1 in a single step. In the other, the cells first transition from level *i* to level *i* − 1 and then reach level *i* + 1 through level *i*. Using the transition probability matrices *P*_*i*_ and *Q*_*i*_, we obtain the recurrence formula as follows:

$$\begin{array}{c} F_{i}=P_{i}+Q_{i}F_{i-1}F_{i}\;\left(i=2,3,\cdots ,M\right) \end{array}$$ (6)

Thus, we have

$$\begin{array}{c} F_{i}={\left(I_{i+1}-Q_{i}F_{i-1}\right)}^{-1}P_{i} \end{array}$$ (7)

where *F*_1_ = *P*_1_, and *I*_*i*+1_ is the (*i* + 1) × (*i* + 1) identity matrix. When the total number of cells reaches *M*, the probability distribution ***π***_***M***_ is calculated by the following formula:

$$\{\begin{array}{ll} \pi_{2}=\left(1,0\right)F_{1} & \\ \pi_{i+1}=\pi_{i}F_{i} & \left(i=3,4,\cdots ,M-1\right) \end{array}$$

Here, *F*_*i*_ is an *i* × (*i* + 1) matrix. Thus, the complexity of calculation is much lower in this case than in the case of the fundamental matrix.

### Approximate aggregation

The algorithm proposed in the previous section still includes the inverse of the (*M* − 1) × *M* matrix. We propose an approximate aggregation to further reduce the complexity of the calculation.

**Fig 1:**
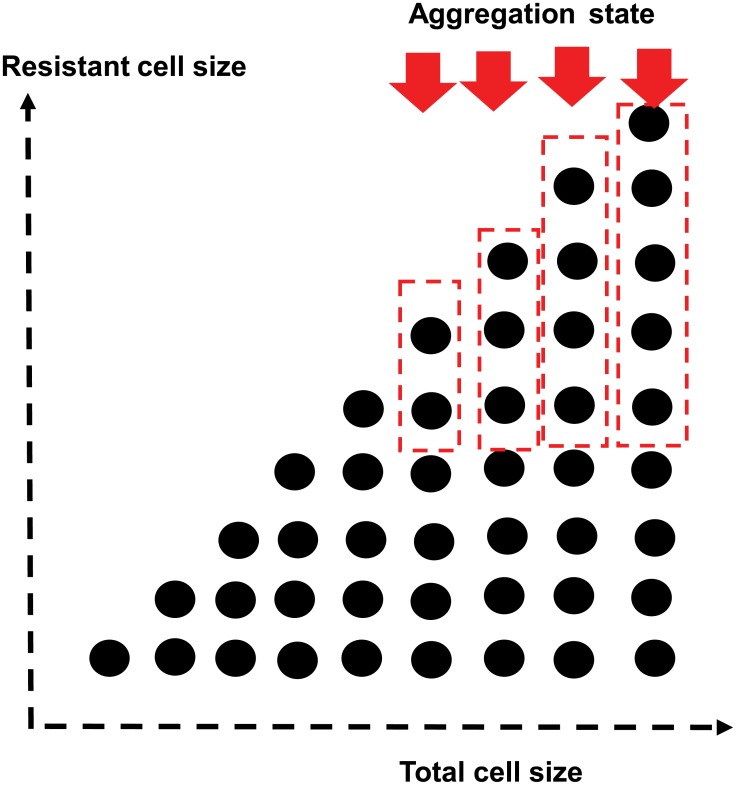
Aggregation. The states that the number of resistant cell is greater than the aggregation size, the states are aggregated to one. In the Fig 1, the states surrounded by dotted line are agrregated to one state.

If the level of states is greater than *m*, we aggregate the states in which the number of resistant cells is greater than *m* to a single state (Fig 1). The level *m* + *k* is the set of states as follows:

$$\begin{array}{c} \left\{(m+k,0),(m+k,1),\ldots ,(m+k,m-1),(m+k,m),\ldots ,(m+k,m+k)\right\} \end{array}$$

From among these, we aggregate the states (*m* + *k*, *m*), (*m* + *k*, *m* + 1), …, (*m* + *k*, *m* + *k*) to one state and denote the aggregated state by (*m* + *k*, m*). Then, state (*m* + *k*, m*) can transit to (*m* + *k* + 1, m*), (*m* + *k* − 1, m*), or (*m* + *k* − 1, *m* − 1).

Let us denote the rate of transition from state (*m* + *k*, m*) to each of state (*m* + *k* + 1, m*), (*m* + *k* − 1, m*), and (*m* + *k* − 1, *m* − 1) by $R_{A}^{k}$, $R_{B}^{k}$, and $R_{C}^{k}$, respectively. These rates can now be expressed as follows:

$$\begin{array}{cc} R_{A}^{k} & =\sum_{x=0}^{k}\{(k-x)\lambda (1-\gamma )+(k-x)\lambda \gamma +(m+x)\alpha \}\times \frac{\pi^{t}(m+k,m+x)}{\sum_{v=0}^{k}\pi^{t}(m+k,m+v)}\\ & =[\frac{1}{2}k(k+1)\lambda +\{(k+1)m+\frac{1}{2}k(k+1)\}\alpha ]\times \frac{\pi^{t}(m+k,m+x)}{\sum_{v=0}^{k}\pi^{t}(m+k,m+v)} \end{array}$$ (8)

$$\begin{array}{cc} R_{B}^{k} & =\sum_{y=0}^{k}\{(k-y)\mu \times \frac{\pi^{t}(m+k,m+x)}{\sum_{v=0}^{k}\pi^{t}(m+k,m+v)}+\sum_{y=1}^{k}\{(m+y)\beta \times \frac{\pi^{t}(m+k,m+x)}{\sum_{v=0}^{k}\pi^{t}(m+k,m+v)}\\ & =[\frac{1}{2}k(k+1)\mu +\{(km+\frac{1}{2}k(k+1)\}\beta ]\times \frac{\pi^{t}(m+k,m+x)}{\sum_{v=0}^{k}\pi^{t}(m+k,m+v)} \end{array}$$ (9)

$$R_{C}^{k}=m\beta \times \frac{\pi^{t}(m+k,m+x)}{\sum_{v=0}^{k}\pi^{t}(m+k,m+v)}$$ (10)

Here, we assume that the following probabilities are the same:

$$\begin{array}{c} \pi^{t}\left(m+k,m\right)=\pi^{t}\left(m+k,m+1\right)=\cdots =\pi^{t}\left(m+k,m+k\right)=\frac{1}{k+1} \end{array}$$ (11)

Now we have

$$\begin{array}{cc} R_{A}^{k} & =\sum_{x=0}^{k}\{(k-x)\lambda (1-\gamma )+(k-x)\lambda \gamma +(m+x)\alpha \}\times \frac{1}{k+1}\\ & =[\frac{1}{2}k(k+1)\lambda +\{(k+1)m+\frac{1}{2}k(k+1)\}\alpha ]\times \frac{1}{k+1} \end{array}$$ (12)

$$\begin{array}{cc} R_{B}^{k} & =\sum_{y=0}^{k}\{(k-y)\mu \times \frac{1}{k+1}+\sum_{y=1}^{k}\{(m+y)\beta \times \frac{1}{k+1}\\ & =[\frac{1}{2}k(k+1)\mu +\{(km+\frac{1}{2}k(k+1)\}\beta ]\times \frac{1}{k+1} \end{array}$$ (13)

$$R_{C}^{k}=m\beta \times \frac{1}{k+1}$$ (14)

Using the sum of the rates *Γ*(*k*), which is described as

$$\begin{array}{cc} \Gamma (k) & =R_{A}^{k}+R_{B}^{k}+R_{C}^{k}\\ & =[\frac{1}{2}k(k+1)(\lambda +\mu )+\{(k+1)m+\frac{1}{2}k(k+1)\}(\alpha +\beta )], \end{array}$$ (15)

we can obtain the probabilities of transition from state (*m* + *k*, m*) to each of (*m* + *k* + 1, m*), (*m* + *k* − 1, m*), and (*m* + *k* − 1, *m* − 1) through the following equations:

$$\begin{array}{c} \frac{R_{A}^{k}}{\Gamma (k)}=\frac{k\lambda +(2m+k)\alpha }{k(\lambda +\mu )+(2m+k)(\alpha +\beta )} \end{array}$$ (16)

$$\begin{array}{c} \frac{R_{B}^{k}}{\Gamma (k)}=\frac{k(k+1)\mu +\{2km+k(k+1)\}\beta }{\left(k+1)(\lambda +\mu )+\{2m(k+1)+k(k+1)(\alpha +\beta )\right\}} \end{array}$$ (17)

$$\begin{array}{c} \frac{R_{C}^{k}}{\Gamma (k)}=\frac{2m\beta }{\left(k+1)(\lambda +\mu )+\{2m(k+1)+k(k+1)(\alpha +\beta )\right\}} \end{array}$$ (18)

After the aggregation, the transition probability submatrix from level *m* + *k* to level *m* + *k* + 1, and from level *m* + *k* to level *m* + *k* − 1, which are denoted by ${\tilde{P}}_{m+k}$ and ${\tilde{Q}}_{m+k}$, respectively, become:

$$\begin{array}{c} {\tilde{P}}_{m+k}=(\begin{array}{ccccccc} \frac{i\lambda (1-\gamma )}{\Gamma_{i0}} & \frac{i\lambda \gamma }{\Gamma_{i0}} & 0 & \cdots & \cdots & \cdots & 0\\ 0 & \frac{\left(i-1)\lambda (1-\gamma \right)}{\Gamma_{i1}} & \frac{(i-1)\lambda \gamma +\alpha }{\Gamma_{i1}} & 0 & \cdots & \cdots & 0\\ 0 & 0 & \frac{\left(i-2)\lambda (1-\gamma \right)}{\Gamma_{i2}} & \frac{(i-2)\lambda \gamma +2\alpha }{\Gamma_{i2}} & \cdots & \cdots & 0\\ \cdots \cdots \cdots \cdots \cdots \cdots \cdots \cdots \cdots \cdots \\ 0 & 0 & 0 & \cdots & \cdots & \frac{\lambda (1-\gamma )}{\Gamma_{ii-1}} & \frac{\lambda \gamma +(i-1)\alpha }{\Gamma_{ii-1}}\\ 0 & 0 & \cdots \cdots \cdots \cdots & \cdots & 0 & \frac{R_{A}^{k}}{\Gamma (k)} \end{array}) \end{array}$$

$$\begin{array}{c} {\tilde{Q}}_{m+k}=(\begin{array}{cccccc} \frac{i\mu }{\Gamma_{i0}} & 0 & 0 & \cdots & \cdots & 0\\ \frac{\beta }{\Gamma_{i1}} & \frac{(i-1)\mu }{\Gamma_{i1}} & 0 & \cdots & \cdots & 0\\ 0 & \frac{2\beta }{\Gamma_{i2}} & \frac{(i-2)\mu }{\Gamma_{i2}} & \cdots & \cdots & 0\\ \cdots \cdots \cdots \cdots \cdots \cdots \cdots \\ 0 & 0 & 0 & \cdots & \frac{(i-1)\beta }{\Gamma_{ii-1}} & 0\\ 0 & 0 & \cdots \cdots & \frac{R_{C}^{k}}{\Gamma (k)} & \frac{R_{B}^{k}}{\Gamma (k)} \end{array}) \end{array}$$

where ${\tilde{P}}_{m+k}$ and ${\tilde{Q}}_{m+k}$ are (*m* + *k*) × (*m* + *k*) matrices. Then we can approximately calculate *F*_*i*_ (*i* = *m*, *m* + 1, *m* + 2, ⋯) using

$$\begin{array}{c} F_{i}={\left(I_{i+1}-{\tilde{Q}}_{i}F_{i-1}\right)}^{-1}{\tilde{P}}_{i} \end{array}$$ (19)

The largest size of the matrix *F*_*i*_ is (*m* + 1) × (*m* + 1), so we can obtain the probability more easily than through exact analysis.

## Results

In this section, we present the numerical results. First, we computed the emerging probabilities of resistance by exact analysis and approximate aggregation. Then we computed relative errors between these numerical results to evaluate the accuracy of approximation. Numerical results computed by the formula in previous study [11] were also compared with the approximation. Second, we computed the emerging probabilities by our approximation method when the detection size was over 10000. Computer simulation was performed to compare the results with approximation. The averages and the 95% confidential intervals of simulation results were computed. Last, we examined the effect of parameter change on emerging probabilities of resistance.

### Exact analysis v.s. aggregate approximation

**Fig 2:**
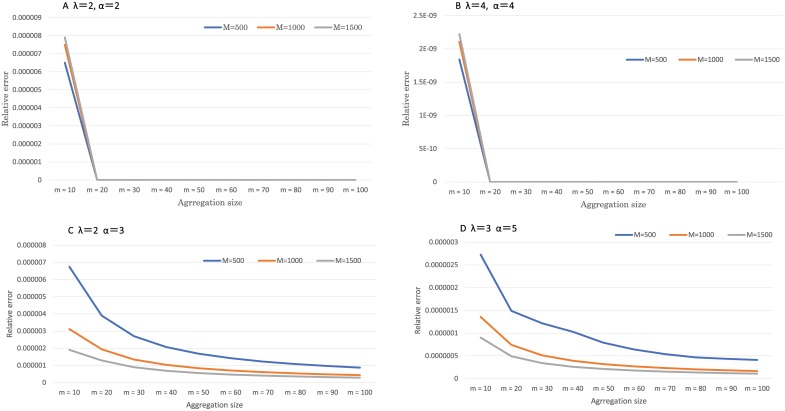
Exact analysis and aggregate approximation. Fig 2 shows the relationship between aggregation size and relative error of emerging probabilities of resistance. The total cell sizes were *M* = 500, 1000 and 1500. The parameter value of *α* and λ were A: *α* = 2.0, λ = 2.0, B: *α* = 4.0, λ = 4.0, C: *α* = 2.0, λ = 3.0, D: *α* = 3.0, λ = 5.0. The parameter value of *β* and *μ* were fixed 1.0 and 10^−5^.

**Fig 3:**
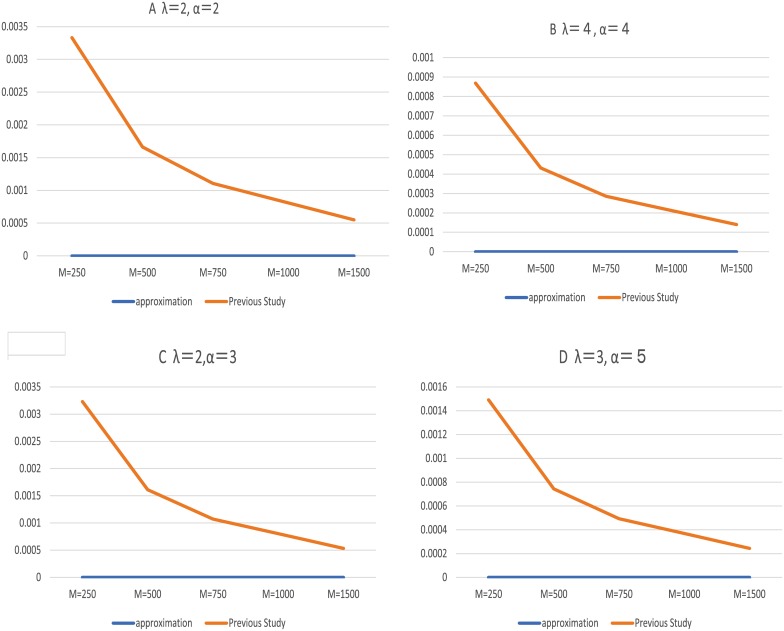
Previous study [11] and aggregate approximation. Fig 3 shows the relationship between the computed values of previous study and relative error when the emerging probabilities of resistance were computed. The total cell size were *M* = 500, 1000 and 1500. The parameter value of *α* and λ were A: *α* = 2.0, λ = 2.0, B: *α* = 4.0, λ = 4.0, C: *α* = 2.0, λ = 3.0, D: *α* = 3.0, λ = 5.0. The parameter value of *β* and *μ* were fixed 1.0 and 10^−5^.

Fig 2 shows the results of emerging probabilities of resistance using the exact analysis method and the aggregation approximation method. We set the detection size M at 500, 1000, and 1500 because the exact analysis took a considerable time when M was greater than 1500. We computed each relative error at variable aggregation size; *m* = 10 ⋯ 100. Computations using the aggregate approximation and the formula in previous study. [11] were executed and computed the relative errors. The relative errors of approximation method were no lower than 10^−6^ order regardless of aggregation size. For comparison, the relative error of the previous study. [11] was no less than the order of 10^−4^. The relative error became smaller as the detection size becomes larger because they regarded the number of cells as continuous valuables when they calculated the probability of resistance. These results are shown in Figs 2 and 3 and Supporting information S1 Table.

#### Selection of aggregation size

To select the aggregation size, we considered following three points. The first one is to reduce computational complexity of large dimension. The execution time becomes longer as the computational complexity of dimension becomes larger. The second, to select the certain small size of cells that the extinction of resistant cells happens rarely enough to have a good approximation of probability of resistance. The third, the accuracy of numerical results.

In order to obtain the probability of extinction of the resistant cells, we neglected the mutation of sensitive cells, which generated resistant cells, and simply considered the birth and death process of resistant cells. This simplified process of resistant cells were regarded as a one-dimensional random walk with absorption walls at sizes 0 and *M*, where *M* was the detection size. If the process started from the aggregation size *m*, the probability of extinction of the resistant cells, *P*_*extinc*_, was as follows:

$$\begin{array}{c} P_{extinc}=\frac{{\left(\frac{\beta }{\alpha }\right)}^{M}-{\left(\frac{\beta }{\alpha }\right)}^{m}}{{\left(\frac{\beta }{\alpha }\right)}^{M}-1} \end{array}$$

When *M* was much larger than *m*, the probability of extinction of the resistant cell was approximated to ${\tilde{P}}_{extinc}=(\frac{\beta }{\alpha }{)}^{m}$. We set *α* = 1.1, *β* = 1.0 according to the previous study, and obtained the following:

$$\begin{array}{c} {\tilde{P}}_{extinc}=\{\begin{array}{cc} \;3.85543\times 10^{-1} & \;(\text{m}=10)\\ \;8.51855\times 10^{-4} & \;(\text{m}=50)\\ \;7.25657\times 10^{-5} & \;(\text{m}=100) \end{array} \end{array}$$

These observations demonstrated that *m* = 100 is appropriate to neglect the probability of extinction of resistant cells, which resulted in a good approximation of the probability of resistance. This was also shown by the numerical results in Fig 2 and Supporting information S1 Table.

#### Computer simulation v.s. aggregate approximation

**Fig 4:**
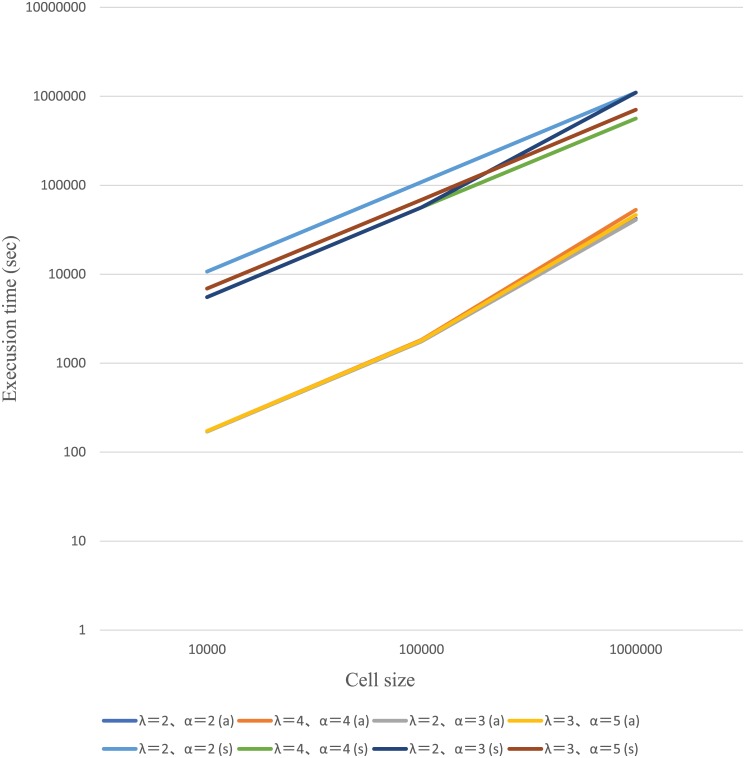
Execution time of simulation and aggregate approximation. Fig 4 shows the execution time for computing approximation values and performing simulation runs. *α* = 2.0, λ = 2.0(a) *α* = 4.0, λ = 4.0(a), *α* = 2.0, λ = 3.0(a), *α* = 3.0, λ = 5.0(a) are execution time of approximation when the detection sizes are *M* = 10000, 100000, 1000000 and *α* = 2.0, λ = 2.0(s), *α* = 4.0, λ = 4.0(s), *α* = 2.0, λ = 3.0(s), *α* = 3.0, λ = 5.0(s) are execution time of simulation runs when the detection sizes are *M* = 10000, 100000, 1000000. The data are plotted on the double logaristhmic coordinates graph.

Next, we computed the emerging probability of resistance for large detection size; *M* = 10000, 30000, 50000, 100000, 1000000. The aggregation size was fixed *m* = 100. We executed computer simulation to confirm the numerical results of approximation. The average and the 95% confidential interval of each computer simulation result showed that the numerical results of approximation fell between the upper level and the lower level of 95% confidential intervals. The execution time of simulation was much longer than those of the approximation. The execution times went a linear increase as larger detection size, and the slope of numerical results of simulation was over 10 times than that of approximations. These results are shown in Fig 4, S2 and S3 Tables.

### Effects of parameter change

#### Division rate and death rate

We examined the effects of parameter change on emerging probabilities of resistance. Table 1 showed the emerging probabilities of resistance at various rates of λ/*μ* and *α*/*β*. The detection size and mutation rate were fixed at 10000 and 10^−5^, respectively.

**Table 1:** Probabilities of resistance depending on the division rate and death rate.

	λ/*μ* = 1.5 λ = 1.5, *μ* = 1.0	λ/*μ* = 2.0 λ = 2.0, *μ* = 1.0	λ/*μ* = 3.0 λ = 3.0, *μ* = 1.0
*α*/*β* = 1.5 *α* = 1.5, *β* = 1.0	1.519021 × 10^−1^	1.2152862 × 10^−1^	1.062601 × 10^−1^
*α*/*β* = 2.0 *α* = 2.0, *β* = 1.0	1.705002 × 10^−1^	1.294396 × 10^−1^	1.094218 × 10^−1^
*α*/*β* = 3.0 *α* = 3.0, *β* = 1.0	1.946913 × 10^−1^	1.410605 × 10^−1^	1.145291 × 10^−1^

*M* = 10000, *γ* = 10^−5^

As λ/*μ* decreased, the probability of resistance increased. This was because as λ/*μ* decreased, a considerable number of divisions was required for the number of cells to reach detection size. As the number of divisions increased, the opportunity to generate resistant cells from sensitive cells increased, thereby increasing the probability of resistance.

When *α*/*β* increased at constant λ/*μ*, the probability of resistance increased. This was in line with the increase in the probability of survival of resistant cells that were generated.

Our observations from our numerical results showed that the probability of resistance was highly dependent on the number of divisions until the cell population reached the detection size.

#### Mutation rate

We computed the probabilities of resistance when the mutation rates changed *γ* = 10^−4^, 10^−5^, and 10^−6^. The numerical results are shown in Table 2. The emerging probabilities of resistance were decreased as the mutation rate smaller.

**Table 2:** Probabilities of resistance depending on mutation rate.

size	parameter	*γ* = 10^−4^	*γ* = 10^−5^	*γ* = 10^−6^
10000	λ = 2.0, *α* = 2.0	7.499780 × 10^−1^	1.294396 × 10^−1^	0.137662 × 10^−1^
	λ = 4.0, *α* = 4.0	6.836007 × 10^−1^	1.086970 × 10^−1^	0.114411 × 10^−1^
	λ = 2.0, *α* = 3.0	7.814099 × 10^−1^	1.410605 × 10^−1^	0.150907 × 10^−1^
	λ = 3.0, *α* = 5.0	7.250626 × 10^−1^	1.211299 × 10^−1^	0.128288 × 10^−1^

*M* = 10000, *μ* = 1.0, *β* = 1.0

## Discussion

In this study, we modeled the cell progression process by a two-dimensional Markov process that was characterized by the total number of cells and the number of resistant cells. We calculated the emerging probability of resistance when the total number of cells reached detection size M, starting from one drug sensitive cell. This probability was equivalent to the probability of being absorbed in the absorbing state wherein the total number of cells was M. To calculate this probability, we needed to inverse matrix of size (*M* + 2)(*M* − 1)/2 × (*M* + 2)(*M* − 1)/2 and the complexity of calculation was *O*(*M*^6^).

We employed the embedded Markov analysis approach and observed only the timepoint at which the total number of cells change. We also derived the recurrence formula for state transition probabilities of the first visit to the set of states wherein the total number of cells was *n* + 1 from the set of states wherein the total number of cells was n. Using this approach and the formula, we proposed an efficient calculation method for emerging probabilities of resistant cells that required *M* times calculation of the (*M* + 1) × (*M* + 1) inverse matrix only. Then, we calculated the emerging probabilities of resistance when the number of cells reached *M* = 1000. However, it took significant execution time for realistic detection sizes such as *M* = 10000 or 100000; thus, we designed a more practical method for calculation.

The approximation approaches inverting a matrix of large dimension have been intensively studied, and this approach may be useful to reduce the execution time for emerging probabilities of resistance. However, as shown above, once the number of resistant cells reached 100, the probability of extinction of resistant cells is under 10^−5^, the information of probability distribution of over 100 resistant cells was not very valuable from the viewpoint of treatment strategy. Hence, we aggregated the states (*m* + *k*, *m*), (*m* + *k*, *m* + 1), … ., (*m* + *k*, *m* + *k*) to a single state for each *k*(*k* = 1, 2, …, *M* − *m*). This approximation method required computing M times calculation of (*m* + 1) × (*m* + 1) inverse matrix to obtain the approximate solutions for the emerging probabilities of resistant cells. We set *m* = 100 in the numerical analysis, because the calculation was completed in a practical execution time for a realistic detection size such as100000.

The numerical analysis showed that approximation errors of emerging probability are negligibly small when *M* = 500 and *M* = 1000 and that our approximation method demonstrated the same level of accuracy when *M* = 1000 or the higher level of accuracy when *M* = 500 compared to the result of a previous study. We also performed a stochastic computer simulation to confirm the results of the approximation method. The simulation performed 1000000 × 10 runs to obtain a 95% confidence interval of the emerging probability of resistance. The results of emerging probability of resistance by the approximation method fell within the 95% confidence interval, and the execution time of our approximation method was considerably shorter than that obtained for our simulation.

The numerical results demonstrated that the probability of resistance was chiefly dependent on the number of cell divisions until the cell population reached the detection size. It is reasonable as drug-resistant cells are generated by mutation in the process of cell division. A large population of cancer cells would have a greater likelihood of generating resistant cells via mutation. As only a few resistant cells exist in the early stage of cancer, if any, the possibility of extinction of resistant cells owing to natural causes cannot be disregarded.

The proposed method in this paper was able to track the transition of cell size. By applying this method, we would be able to follow the transition of the number of cells under drug administration. The next step in our study is to design the treatment strategy based on the analysis under drug administration.

In many cases, multiple different mutations can confer resistance and the mutagenic processes leading to such mutations may be different. Then, clones may have different growth and death rates in accordance depending on the types of mutations. It is of interest to compute the number of resistant cells of multiple types when the cell population reaches the detection size. However, it would be more difficult to extend the framework in this paper to the case of multiple resistance mutations. For example, it is necessary to analyze a three-dimensional Markov process in the case of two types of mutations. Thus, another effective approximation would be necessary for the analysis.

## Supporting information

S1 File Source code. (PDF)

S1 Table Exact analysis, previous study and aggregate approximation. (PDF)

S2 Table Simulation and aggregate approximation. (PDF)

S3 Table Execution time. (PDF)
