# Supplementary material for: A numerical approach for a discrete Markov model for progressing drug resistance of cancer
Source: PLoS Comput Biol. 2019 Feb 19;15(2):e1006770. doi: 10.1371/journal.pcbi.1006770 (PMC6396936; doi:10.1371/journal.pcbi.1006770)
Supplement: S1 File — (PDF) [file pcbi.1006770.s001.pdf]

```
/* Aggregation Approximation */

#include <stdio.h>
#include <stdlib.h>
#include <math.h>


#define N 10000 /* Detection size */
#define M 100 /* Aggregation size */

#define λ 2.0 /* Parameter: division rate (sensitive) */
#define μ 1.0 /* Parameter: death rate (sensitive) */
#define α 3.0 /* Parameter: division rate (resistant) */
#define β 1.0 /* Parameter: death rate (resistant) */
#define γ 0.00001 /* Parameter: mutation rate */

int num,i,j;

double **dmatrix(int nr1, int nr2, int nl1, int nl2); /* Memory allocation */
void free_dmatrix(double **a, int nr1, int nr2, int nl1, int nl2); /* Memory release */

void up_matrix (double **a, char c, FILE *fout); /* Function for calculating matrix (exact): $P_i$ */
void dn_matrix (double **b, char c, FILE *fout); /* Function for calculating matrix (exact): $Q_i$ */
void en_matrix (double **e, char c, FILE *fout); /* Function for calculating unit matrix (exact): $I_i$ */
void product1 (double **c, double **b, double **f, double **e, double **sa, FILE *fout); /* Function for calculating (exact): $I_{i+1} – Q_iF_{i-1}$*/
void gauss (double **sa, double **inv_sa, FILE *fout); /* Function for calculating (exact): $(I_{i+1} – Q_iF_{i-1} )^{-1}$ */
void product2 (double **inv_sa, double **a, double **g, FILE *fout); /* Function for calculating (exact) : $F_i = ( I_{i+1} – Q_iF_{i-1} )^{-1}P_i$ */
void product3 (double **d, double **D, double **g, FILE *fout); /* Function for calculating (exact) : $\pi_{i+1} = (1,0) F_1 F_2 F_3…F_{i-1} F_i$ */
void product4 (double **dinit, double **g, double **d, FILE *fout);/* Function for calculating (exact) : $\pi_2 = (1,0) F_1$ */

void upabs_matrix( double **a_abs, char c, FILE *fout);    /* Function for calculating matrix (aggregation) : $\tilde{P}_i$ */
void dnabs_matrix( double **b_abs, char c, FILE *fout); /* Function for calculating matrix (aggregation) :  $\tilde{Q}_i$ */
void enabs_matrix( double **e_abs, char c, FILE *fout); /* Function for calculating matrix (aggregation) :  $I_i$ */
void product1_abs ( double **c_abs, double **b_abs, double **f_abs, double **e_abs, double **sa_abs, FILE *fout);/*Function for calculating (aggregation):$\tilde{I}_{i+1}$ -  $\tilde{Q}_i\tilde{F}_{i-1}$*/
void gauss_abs( double **sa_abs, double **inv_saabs,FILE *fout); /*Function for calculating (aggregation): $(\tilde{I}_{i+1} –  \tilde{Q}_i\tilde{F}_{i-1} )^{-1}$ */
```

```
void product2_abs( double **inv_saabs, double **a_abs,double **g_abs,FILE *fout); /* Function for calculating (aggregation) : $\tilde{F}_i = (\tilde{I}_{i+1} - \tilde{Q}_i\tilde{F}_{i-1})^{-1} \tilde{P}_i$*/
void product3_abs( double **d_abs,    double **D_abs, double **g_abs,FILE *fout); /* Function for calculating (aggregation) : $\tilde{\pi}_{i+1} = (1,0) \tilde{F}_1 \tilde{F}_2 \tilde{F}_3 \ldots \tilde{F}_{i-1} \tilde{F}_i$ */

void pre_product2( double **inv_sa, double **a_abs,double **g_abs,FILE *fout);/* Function for calculating (aggregation point) : $\tilde{F}_i = (\tilde{I}_{i+1} - \tilde{Q}_i\tilde{F}_{i-1})^{-1} \tilde{P}_i$ */
void pre_product3( double **d_abs,    double **D, double **g_abs,FILE *fout); /* Function for calculating (aggregation point) : $\tilde{\pi}_{i+1} = (1,0) \tilde{F}_1 \tilde{F}_2 \tilde{F}_3 \ldots \tilde{F}_{i-1} \tilde{F}_i$*/

double keisan (double **x, double *prob, double *prob_d, char c, FILE *fout); /* Function for calculating probability */


/* Main program */
int main (void)
{
  FILE *fout;
  double **a, **b, **e, **c, **f, **g, **d, **D, **sa, **inv_sa, **dinit;
  double **a_abs, **b_abs, **e_abs, **c_abs, **f_abs, **g_abs, **d_abs, **D_abs, **sa_abs, **inv_saabs;

/* Initial values setting */
double sum1=0.0;
double sum2=0.0;
double prob=0.0;
double prob_d=0.0

/* File open */
  if((fout=fopen("PROBABILITY_AAA_aa.txt","w")) = NULL)
  {
          printf("cannot create the file  ：PROBABILITY_AAA_aa.txt¥n");
          exit (1);
  }

for (num = =1; num<=N; num++) {

/* Calculation: π2 and emerging probability of resistance */
    if (num = =1) {

/* Memory allocation */
```

```
  a = dmatrix(1, num+1, 1, num+2);
  g = dmatrix(1, num+1, 1, num+2);
  d = dmatrix(1, 1, 1, num+2);
  dinit = dmatrix(1, 1, 1, 2);

/* Pi */
  up_matrix(a,'G',fout);
  for (i=1; i<=num+1 ;i++){
                  for (j=1; j<=num+2; j++) {
                  g[i][j] = a[i][j];
                  }
  }
/*Calculation: π2 = (1,0) F1 */
product4(dinit, g, d, fout);

/* Probability of resistance */
keisan(dint, prob, prob_d, P, *fout);

/* Print out */
fprintf (fout,"Probability is: ¥n",c);
fprintf (fout,"%12.10f¥t", keisan(d,'P', fout));
fprintf (fout,"¥n");


/* Memory release */
  free_dmatrix( a, 1, num+1, 1, num+2 );
  free_dmatrix( dinit, 1, 1, 1, 2 );
  }

/* Calculation Before Aggregation (= exact analysis) */
  else if (num>=2 && num<=M)

  {

/* Memory allocation */
```

```
  f = dmatrix(1, num, 1, num+1);
  D = dmatrix(1, 1, 1, num+1);
  for(i=1; i<=num;i++){
                  for (j=1; j<=num+1; j++) {
                  f[i][j] = g[i][j];
                  }
  }

for (i=1; i<=1;i++){
                  for(j=1; j<=num+1;j++){
                  D[i][j] = d[i][j];
                  }
  }

/* Memory release */
  free_dmatrix (g, 1, num, 1, num+1);
  free_dmatrix (d, 1, 1, 1, num+1);

/* Memory allocation */
  a = dmatrix (1, num+1, 1, num+2);
  b = dmatrix (1, num+1, 1, num);
  c = dmatrix (1, num+1, 1, num+1);
  e = dmatrix (1, num+1, 1, num+1);
  g = dmatrix (1, num+1, 1, num+2);
  sa = dmatrix (1, num+1, 1, num+1);
  inv_sa = dmatrix(1, num+1, 1, num+1);
  d =dmatrix(1, 1, 1, num+2);

/* Matrix setting: P_i, Q_i, I_i */
up_matrix(a,'A',fout);
dn_matrix(b,'B',fout);
en_matrix(e,'I',fout);

/* Calculation: π_{i+1} = (1,0) F_1 F_2 F_3…F_{i-1} F_i */
product1(c,b,f,e,sa,fout);    /* I_{i+1} – Q_iF_{i-1} */
```

```
gauss(sa,inv_sa,fout);    /* (Ii+1 – QiFi-1 )-1 */
product2(inv_sa,a,g,fout);    /* Fi = ( Ii+1 – QiFi-1 )-1Pi */
product3(d,D,g,fout);    /* πi+1 = (1,0) F1 F2 F3…Fi-1 Fi */

/* Probability of resistance */
keisan(d_abs, prob, prob_d, P, *fout);

/* Print out */
fprintf( fout,"Probability is : ¥n",c);
fprintf( fout,"%12.10f¥t",keisan(d,'P', fout));
fprintf( fout,"¥n");


/* Memory release */
free_dmatrix (a, 1, num+1, 1, num+2);
free_dmatrix (b, 1, num+1, 1, num);
free_dmatrix (c, 1, num+1, 1, num+1);
free_dmatrix (e, 1, num+1, 1, num+1);
free_dmatrix (f, 1, num, 1, num+1);
free_dmatrix (sa, 1, num+1, 1, num+1);
free_dmatrix (inv_sa, 1, num+1, 1, num+1);
free_dmatrix (D, 1, 1, 1, num+1);
  }

/* Calculation Aggregation Point */
else if (num = = M+1)

  {

/* Memory allocation */
  f = dmatrix(1, num, 1, num+1);
  D = dmatrix(1, 1, 1, num+1);
  for(i=1;i<=num;i++){
                  for(j=1;j<=num+1;j++){
                  f[i][j] = g[i][j];
```

```
                  }
  }

for(i=1;i<=1;i++){
                  for(j=1;j<=num+1;j++){
                  D[i][j] = d[i][j];
                  }
  }

/* Memory release */
  free_dmatrix (g, 1, num, 1, num+1);
  free_dmatrix (d, 1, 1, 1, num+1);

/* Memory allocation */
  a_abs = dmatrix (1, M+2, 1, M+2);
  b = dmatrix (1, num+1, 1, num);
  c = dmatrix (1, num+1, 1, num+1);
  e = dmatrix (1, num+1, 1, num+1);
  g_abs = dmatrix (1, M+2, 1, M+2);
  sa = dmatrix(1, num+1, 1, num+1);
  inv_sa = dmatrix (1, num+1, 1, num+1);
  d_abs = dmatrix( 1, 1, 1, M+2);

/* Matrix setting:  P̃_i, Q_i, I_i */
upabs_matrix (a_abs,'A',fout);
dn_matrix (b,'B',fout);
en_matrix(e,'I',fout);

/* Calculation: π_{i+1} = (1,0) F_1 F_2 F_3…F_{i-1} F_i */
product1(c,b,f,e,sa,fout);    /* I_{i+1} – Q_iF_{i-1} */
gauss (sa,inv_sa,fout);    /* (I_{i+1} – Q_iF_{i-1} )^{-1} */
pre_product2 (inv_sa,a_abs,g_abs,fout);    /* F_i = ( I_{i+1} – Q_iF_{i-1} )^{-1}P_i */
pre_product3 (d_abs,D,g_abs,fout);      /* π_{i+1} = (1,0) F_1 F_2 F_3…F_{i-1} F_i */

/* Probability of resistance */
```

```
keisan(d_abs, prob, prob_d, P, *fout);

/* Print out */
fprintf (fout,"Probability is: ¥n",c);
fprintf (fout,"%12.10f¥t", keisan(d_abs,'P', fout));
fprintf (fout,"¥n");


/* Memory release */
free_dmatrix(a_abs, 1, M+2, 1, M+2 );
free_dmatrix(b, 1, num+1, 1, num);
free_dmatrix(c, 1, num+1, 1, num+1 );
free_dmatrix(e, 1, num+1, 1, num+1 );
free_dmatrix(f, 1, num, 1, num+1 );
free_dmatrix(sa, 1, num+1, 1, num+1 );
free_dmatrix(inv_sa, 1, num+1, 1, num+1 );
free_dmatrix(D, 1, 1, 1, num+1 );
  }


/* Calculation After Aggregation */
else
{

/* Memory allocation */
  f_abs = dmatrix(1, M+2, 1, M+2);
  D_abs = dmatrix(1, 1, 1, M+2);
  For (i=1; i<=M+2;i++){
                  for (j=1; j<=M+2; j++){
                  f_abs[i][j] = g_abs[i][j];
                  }
  }

for(i=1;i<=1;i++){
                  for(j=1;j<=M+2;j++){
```

```
                  D_abs[i][j] = d_abs[i][j];
                  }
  }


/* Memory release */
  free_dmatrix(g_abs, 1, M+2, 1, M+2);
  free_dmatrix(d_abs, 1, 1, 1, M+2);

/* Memory allocation */
  a_abs = dmatrix(1, M+2, 1, M+2);
  b_abs = dmatrix(1, M+2, 1, M+2);
  c_abs = dmatrix(1, M+2, 1, M+2);
  e_abs = dmatrix(1, M+2, 1, M+2);
  g_abs = dmatrix(1, M+2, 1, M+2);
  sa_abs = dmatrix(1, M+2, 1, M+2);
  inv_saabs = dmatrix(1, M+2, 1, M+2);
  d_abs = dmatrix(1, 1, 1, M+2);


/* Matrix setting:  ~P_i,  ~Q_i,  ~I_i */
upabs_matrix(a_abs,'A',fout);
dnabs_matrix(b_abs,'B',fout);
enabs_matrix(e_abs,'I',fout);

/*Calculation:  ~π_{i+1} = (1,0)  ~F_1  ~F_2  ~F_3…~F_{i-1}  ~F_i */
product1_abs(c_abs,b_abs,f_abs,e_abs,sa_abs,fout);    /*  ~I_{i+1} -  ~Q_i~F_{i-1} */
gauss_abs(sa_abs,inv_saabs,fout);    /*    (~I_{i+1} –  ~Q_i~F_{i-1} )^{-1} */
product2_abs(inv_saabs,a_abs,g_abs,fout);    /*~F_i = (~I_{i+1} –  ~Q_i~F_{i-1} )^{-1}  ~P_i */
product3_abs(d_abs,D_abs,g_abs,fout);    /*  ~π_{i+1} = (1,0)  ~F_1  ~F_2  ~F_3…~F_{i-1}  ~F_i */

/* Probability of resistance */
keisan(d_abs, prob, prob_d, P, *fout);

/* Print out */
```

```
fprintf (fout,"Probability is : ¥n",c);
fprintf (fout,"%12.10f¥t",prob);
fprintf (fout,"¥n");


/* Memory release */
free_dmatrix (a_abs, 1, M+2, 1, M+2);
free_dmatrix (b_abs, 1, M+2, 1, M+2);
free_dmatrix (c_abs, 1, M+2, 1, M+2);
free_dmatrix (e_abs, 1, M+2, 1, M+2);
free_dmatrix (f_abs, 1, M+2, 1, M+2);
free_dmatrix (sa_abs,1, M+2, 1, M+2);
free_dmatrix (inv_saabs, 1, M+2, 1, M+2);
free_dmatrix (D_abs, 1, 1, 1, M+2);
  }

}

return (0);
  }

/* Function for calculating matrix (exact): Pi */
void up_matrix (double **a, char c, FILE *fout)
  {
         int i,j;
        for (i=1; i<=num+1; i++)
        {
                for (j=1; j<=num+2;j++)
                {
                        if(I = = j) {
                        a[i][j] = ((num-i+1) *λ*(1-γ))/((λ+μ)*(num-i+1)+(α+β)*(i-1));
                        }
                        else if(i+1==j) {
                        a[i][j] = ((num-i+1)*λ*γ+(i-1)*α)/((λ+μ)*(num-i+1)+(α+β)*(i-1));
                        }
```

```
                        else{
                        a[i][j]=0;
                        }
        }
        }
  }
```

```
/* Function for calculating matrix (exact): Qi */
   void dn_matrix(double **b, char c, FILE *fout)
  {
          int i,j;

        for(i=1;i<=num+1;i++)
        {
                for(j=1;j<=num;j++)
                {
                if(i==j){
                        b[i][j]=((num-i+1)*μ)/((λ+μ)*(num-i+1)+(α+β)*(i-1));
                        }
                        else if(i==j+1){
                        b[i][j]=((i-1)*β)/((λ+μ)*(num-i+1)+(α+β)*(i-1));
                        }
                        else{
                        b[i][j]=0;
                        }
        }
        }
  }
```

```
/* Function for calculating matrix (aggregation) :  P̃i */
void upabs_matrix( double **a_abs, char c, FILE *fout)
  {
          int i,j;
```

```
        for(i=1;i<=M+2;i++)
        {
                for(j=1;j<=M+2;j++)
                {
                        a_abs[i][j]=0.0;
                        a_abs[M+2][M+2]=((num-M)*λ+(2*M+(num-M))*α)/((num-M)*(λ+μ)+(2*M+(num-M))*(α+β));
                }
        }


        for(i=1;i<=M+1;i++)
        {
                for(j=1;j<=M+2;j++)
                {

                if(i==j){
                        a_abs[i][j]=((num-i+1)*λ*(1-γ))/((λ+μ)*(num-i+1)+(α+β)*(i-1));
                        }

                        else if(i+1==j){
                        a_abs[i][j]=((num-i+1)*λ*γ+(i-1)*α)/((λ+μ)*(num-i+1)+(α+β)*(i-1));
                        }

                        else{
                        a_abs[i][j]=0;
                        }
        }
 }
}


/* Function for calculating matrix (aggregation) :  Q̃i */
  void dnabs_matrix(double **b_abs, char c, FILE *fout)
    {
int i,j;
```

```
for(i=1;i<=M+2;i++)
	{
		for(j=1;j<=M+2;j++)
		{

			if(num<=46340)
			{

			b_abs[i][j]=0.0;
			b_abs[M+2][M+2]=((num-M)*(num-M+1)*μ+(2*(num-M)*M+(num-M)*(num-M+1))*β)/((λ+μ)*(num-M)*(num-M+1)+(α+β)*(2*(num-
M+1)*M+(num-M)*(num-M+1)));
			b_abs[M+2][M+1]=(2*M*β)/((λ+μ)*(num-M)*(num-M+1)+(α+β)*(2*(num-M+1)*M+(num-M)*(num-M+1)));
			}

			else
			{
			b_abs[i][j]=0.0;
			b_abs[M+2][M+2]=((num-M)*μ+(2*M*(1-1/(num-M+1))+(num-M))*β)/((λ+μ)*(num-M)+(α+β)*(2*M+(num-M)));
			b_abs[M+2][M+1]=(1/(num-M+1))*(2*β*M)/((λ+μ)*(num-M)+(α+β)*(2*M+(num-M)));
			}
		}
}

for(i=1;i<=M+1;i++)
	{
		for(j=1;j<=M+1;j++)
		{
		if(i==j){
				b_abs[i][j]=((num-i+1)*μ)/((λ+μ)*(num-i+1)+(α+β)*(i-1));
		}

		  else if(i==j+1){
			b_abs[i][j]=(j*β)/((λ+μ)*(num-i+1)+(α+β)*(i-1));
			}
```

```
                                        else{
                                        b_abs[i][j]=0;
                                        }
                        }
                }
 }


/* Function for calculating unit matrix (exact): Ii */
    void en_matrix(double **e, char c, FILE *fout)
 {
          int i,j;
        for(i=1;i<=num+1;i++)
        {
                for(j=1;j<=num+1;j++)
                {
                        if(i==j){
                        e[i][j]=1.0;
                        }
                        else{
                        e[i][j]=0.0;
                        }
        }
        }
 }


/* Function for calculating (exact): Ii+1 – QiFi-1*/
void product1(double **c, double **b, double **f, double **e, double **sa, FILE *fout)
{
  int i, j, k;

for( i = 1 ; i <= num+1 ; i++)
      {
         for( j = 1 ; j <= num+1 ; j++)
```

```
            {
            c[i][j] = 0.0;
            for( k = 1 ; k <= num ; k++)
            {
                        c[i][j] += b[i][k] * f[k][j];
            }
                        sa[i][j] = e[i][j] - c[i][j];

            }
      }

}
```

/* Function for calculating (exact) : $F_i = (I_{i+1} – Q_iF_{i-1})^{-1}$ */

```
void gauss (double **sa, double **inv_sa, FILE *fout)
{
   int i, j, k,ip;
   double alpha, tmp;
   double amax , eps = pow(10.0, -13.0); /* eps = 10 ^ {-13} */

            for(i=1;i<=num+1;i++)
            {
                        for(j=1;j<=num+1;j++)
                        {
                                    if(i==j){
                                    inv_sa[i][j]=1.0;
                                    }
                                    else{
                                    inv_sa[i][j]=0.0;
                                    }
            }
            }
```

```
    for( k=1; k <=num+1; k++)
    {

    /* Pivoting */
    amax = fabs(sa[k][k]);
    ip = k;
    for(i = k+1; i < num+1; i++)
    {
        if( fabs(sa[i][k]) > amax )
        {
            amax = fabs(sa[i][k]);
            ip=i;
}
}

/* Determination of regularity */
if( amax < eps ) printf(" The matrix is not regular ¥n");

/* Exchanging rows */
if( ip != k)
{
for( j = 1; j <= num+1; j++)
{
tmp = sa[k][j] ;
sa[k][j] = sa[ip][j];
sa[ip][j] = tmp;
tmp = inv_sa[k][j];
inv_sa[k][j] = inv_sa[ip][j];
inv_sa[ip][j] = tmp;
}
}

    /* Forward elimination */
                alpha =1.0/sa[k][k];
```

```
            for( i =1;i<= num+1; i++)
                   {
               sa[k][i] = alpha*sa[k][i];
               inv_sa[k][i] = alpha*inv_sa[k][i];
                   }

/* pivot column =0 */
for (i=1;i<=num+1;i++)
{
if (i != k)
{
tmp = sa[i][k];
for (j=1 ; j<=num+1 ; j++)
{
sa[i][j] = sa[i][j]-tmp*sa[k][j];
inv_sa[i][j] = inv_sa[i][j]-tmp*inv_sa[k][j];
}
}
}
   }
}


/* Function for calculating (exact) : F_i = ( I_i+1 – Q_iF_i-1 )^-1P_i */
void product2 (double **inv_sa, double **a,double **g, FILE *fout)
{
   int i, j, k;

for( i = 1 ; i <= num+1 ; i++)
       {
            for( j = 1 ; j <= num+2 ; j++)
                 {
                                   g[i][j]=0.0;
               for( k = 1 ; k <= num+1 ; k++)
                 {
```

```
                    g[i][j] += inv_sa[i][k] * a[k][j];

            }
            }
        }

}
```

/* Function for calculating (exact) : π_{i+1} = (1,0) F_1 F_2 F_3…F_{i-1} F_i */

```
void product3( double **d, double **D, double **g, FILE *fout)
{
   int i, j, k;

for( i = 1 ; i <=1 ; i++)
        {
            for( j = 1 ; j <= num+2 ; j++)
            {
                    d[i][j]=0.0;
            for( k = 1 ; k <= num+1 ; k++)
            {
                    d[i][j] += D[i][k] * g[k][j];

            }
            }
        }

}
```

/* Function for calculating (exact) : π_2 = (1,0) F_1 */

```
void product4(double **dinit, double **g, double **d, FILE *fout)
{
   int i, j, k;
```

```
    dinit[1][1]=1.0;
    dinit[1][2]=0.0;

for( i = 1 ; i <= 1 ; i++)
        {
            for( j = 1 ; j <= num+2 ; j++)
                 {
                                 d[i][j]=0.0;
                 for( k = 1 ; k <= num+1 ; k++)
                 {
                                 d[i][j] += dinit[i][k] * g[k][j];

                 }
                 }
        }

}


* Function for calculating matrix (aggregation) :  Ĩi */
void enabs_matrix( double **e_abs, char c, FILE *fout)
{
                  int i,j;
                 for(i=1;i<=M+2;i++)
                 {
                                 for(j=1;j<=M+2;j++)
                                 {
                                                 if(i==j){
                                                 e_abs[i][j]=1.0;
                                                 }
                                                 else{
                                                 e_abs[i][j]=0.0;
                                                 }
                 }
                 }
```

```
}
```

/*Function for calculating (aggregation): $\tilde{I}_{i+1} - \tilde{Q}_i\tilde{F}_{i-1}$ */

```
void product1_abs(double **c_abs, double **b_abs, double **f_abs, double **e_abs,double **sa_abs,FILE *fout)
{
   int i, j, k;

for( i = 1 ; i <= M+2 ; i++)
       {
          for( j = 1 ; j <= M+2 ; j++)
               {
               c_abs[i][j] = 0.0;
               for( k = 1 ; k <= M+2 ; k++)
               {
                              c_abs[i][j] += b_abs[i][k] * f_abs[k][j];
                              sa_abs[i][j] = e_abs[i][j] - c_abs[i][j];

               }
               }
       }

}
```

/*Function for calculating (aggregation): $(\tilde{I}_{i+1} - \tilde{Q}_i\tilde{F}_{i-1})^{-1}$ */

```
void gauss_abs(double **sa_abs, double **inv_saabs,FILE *fout)
{
   int i, j, k,ip;
   double alpha, tmp;
   double amax , eps = pow(10.0, -13.0); /* eps = 10 ^ {-13} */

               for(i=1;i<=M+2;i++)
               {
                              for(j=1;j<=M+2;j++)
```

```
				{
						if(i==j){
						inv_saabs[i][j]=1.0;
						}
						else{
						inv_saabs[i][j]=0.0;
						}
			}
			}



    for( k=1; k <=M+2; k++)
    {

    /* Pivoting    */
    amax = fabs(sa_abs[k][k]);
    ip = k;
    for(i = k+1; i < M+2; i++)
    {
        if( fabs(sa_abs[i][k]) > amax )
        {
            amax = fabs(sa_abs[i][k]);
            ip=i;
}
}

/* Determination of regularity */
if( amax < eps ) printf(" The matrix is not regular ¥n");

/* Exchanging rows */
if( ip != k)
{
for( j = 1; j <= M+2; j++)
{
```

```
tmp = sa_abs[k][j] ;
sa_abs[k][j] = sa_abs[ip][j];
sa_abs[ip][j] = tmp;
tmp = inv_saabs[k][j];
inv_saabs[k][j] = inv_saabs[ip][j];
inv_saabs[ip][j] = tmp;
}
}

  /* Forward elimination */
                alpha =1.0/sa_abs[k][k];
      for( i =1;i<= M+2; i++)
                {
             sa_abs[k][i] = alpha*sa_abs[k][i];
             inv_saabs[k][i] = alpha*inv_saabs[k][i];
                }

/* Pivot columns=0 */
for (i=1;i<=M+2;i++)
{
if (i != k)
{
tmp = sa_abs[i][k];
for (j=1 ; j<=M+2 ; j++)
{
sa_abs[i][j] = sa_abs[i][j]-tmp*sa_abs[k][j];
inv_saabs[i][j] = inv_saabs[i][j]-tmp*inv_saabs[k][j];
}
}
}
  }
}
```

/* Function for calculating (aggregation) : $\tilde{F}_i = (\tilde{I}_{i+1} - \tilde{Q}_i\tilde{F}_{i-1})^{-1} \tilde{P}_i$*/

```
void product2_abs( double **inv_saabs, double **a_abs,double **g_abs,FILE *fout)
{
   int i, j, k;

for( i = 1 ; i <= M+2 ; i++)
      {
         for( j = 1 ; j <= M+2 ; j++)
              {
                           g_abs[i][j]=0.0;
              for( k = 1 ; k <= M+2 ; k++)
              {
                           g_abs[i][j] += inv_saabs[i][k] * a_abs[k][j];

              }
              }
      }

}
```

/* Function for calculating (aggregation) : $\tilde{\pi}_{i+1} = (1,0)\ \tilde{F}_1\ \tilde{F}_2\ \tilde{F}_3 \ldots \tilde{F}_{i-1}\ \tilde{F}_i$ */

```
void product3_abs( double **d_abs,    double **D_abs, double **g_abs,FILE *fout)
{

   int i, j, k;

for( i = 1 ; i <=1 ; i++)
      {
         for( j = 1 ; j <= M+2 ; j++)
              {
                           d_abs[i][j]=0.0;
              for( k = 1 ; k <= M+2 ; k++)
              {
                           d_abs[i][j] += D_abs[i][k] * g_abs[k][j];

              }
```

```
            }
        }

    }
```

/* Function for calculating (aggregation point) : $\tilde{F}_i = (\tilde{I}_{i+1} - \tilde{Q}_i\tilde{F}_{i-1})^{-1} \; \tilde{P}_i$ */

```
void pre_product2( double **inv_sa, double **a_abs,double **g_abs,FILE *fout)
            {
   int i, j, k;

for( i = 1 ; i <= num+1 ; i++)
        {
            for( j = 1 ; j <= M+2 ; j++)
            {
                        g_abs[i][j]=0.0;
            for( k = 1 ; k <= num+1 ; k++)
            {
                        g_abs[i][j] += inv_sa[i][k] * a_abs[k][j];

            }
            }
        }

}
```

* Function for calculating (aggregation point) : $\tilde{\pi}_{i+1} = (1,0) \; \tilde{F}_1 \; \tilde{F}_2 \; \tilde{F}_3 \ldots \tilde{F}_{i-1} \; \tilde{F}_i$*/

```
void pre_product3( double **d_abs,    double **D, double **g_abs,FILE *fout)
{

   int i, j, k;

for( i = 1 ; i <=1 ; i++)
        {
```

```
        for( j = 1 ; j <= M+2 ; j++)
          {
                    d_abs[i][j]=0.0;
          for( k = 1 ; k <= num+1 ; k++)
          {
                    d_abs[i][j] += D[i][k] * g_abs[k][j];

          }
          }
     }

}


/* Function for calculating probability */
double keisan (double **x, double *prob, double *prob_d, char c, FILE *fout)
  {
int i,j;

if (num<=M+1){
for (i=1;i<=1;i++)
{
for(j=1;j<=num+2;j++){
sum1=sum1+x[i][j];
}
}

for (i=1;i<=1;i++)
{
for (j=2;j<=num+2;j++)
{
sum2=sum2+x[i][j];
}
}
}
```

```
else{
                  for (i=1;i<=1;i++)
{
for(j=1;j<=M+2;j++){
sum1=sum1+x[i][j];
}
}

for (i=1;i<=1;i++)
{
for (j=2;j<=M+2;j++)
{
sum2=sum2+x[i][j];
}
}

}

prob=sum2/sum1;
prob_d=prob_d+prob;

}


/* Memory allocation */
double **dmatrix(int nr1, int nr2, int nl1, int nl2)
{
    int i, nrow, ncol;
    double **a;

    nrow = nr2 - nr1 + 1 ;
    ncol = nl2 - nl1 + 1 ;

    if ( ( a = (double **)malloc( nrow*sizeof(double *) ) ) == NULL )
```

```
    {
        printf("caanot keep memories (Matrix a)¥n");
        exit(1);
    }
    a = a - nr1;

    for( i=nr1; i<=nr2; i++) a[i] = (double *)malloc(ncol*sizeof(double));
    for( i=nr1; i<=nr2; i++) a[i] = a[i]-nl1;

    return(a);
}


/* Memory release */
void free_dmatrix(double **a, int nr1, int nr2, int nl1, int nl2)
{
    int i;

    for ( i = nr1 ; i <= nr2 ; i++) free((void *)(a[i]+nl1));
    free((void *)(a+nr1));
}
```
