# Supplementary material for: A numerical approach for a discrete Markov model for progressing drug resistance of cancer
Source: PLoS Comput Biol. 2019 Feb 19;15(2):e1006770. doi: 10.1371/journal.pcbi.1006770 (PMC6396936; doi:10.1371/journal.pcbi.1006770)
Supplement: S1 Table — (PDF) [file pcbi.1006770.s002.pdf]

M=500

parameter	method	probability	relative error
λ =2、 α =2	exact	0.00689604	—
	previous study	0.00690751	0.001663
	approximation		
	m = 10	0.00689608	6.50E-06
	m = 20	0.00689604	8.16E-10
	m = 30	0.00689604	2.03E-13
	m = 40	0.00689604	0
	m = 50	0.00689604	0
	m = 60	0.00689604	0
	m = 70	0.00689604	0
	m = 80	0.00689604	0
	m = 90	0.00689604	0
	m = 100	0.00689604	0
λ =4、 α =4	exact	0.00573465	—
	previous study	0.00573712	0.000431
	approximation		
	m = 10	0.00573465	1.84E-09
	m = 20	0.00573465	0
	m = 30	0.00573465	0
	m = 40	0.00573465	0
	m = 50	0.00573465	0
	m = 60	0.00573465	0
	m = 70	0.00573465	0
	m = 80	0.00573465	0
	m = 90	0.00573465	0
	m = 100	0.00573465	0
λ =2、 α =3	exact	0.00756244	—
	previous study	0.00757463	0.001612
	approximation		
	m = 10	0.00756239	6.74E-06
	m = 20	0.00756241	3.90E-06
	m = 30	0.00756242	2.70E-06
	m = 40	0.00756242	2.07E-06
	m = 50	0.00756243	1.68E-06
	m = 60	0.00756243	1.42E-06
	m = 70	0.00756243	1.23E-06
	m = 80	0.00756243	1.08E-06
	m = 90	0.00756243	9.66E-07
	m = 100	0.00756243	8.73E-07
λ =3、 α =5	exact	0.00643056	—
	previous study	0.00643534	0.000743
	approximation		
	m = 10	0.00643054	2.72E-06
	m = 20	0.00643055	1.49E-06
	m = 30	0.00643055	1.22E-06
	m = 40	0.00643056	1.03E-06
	m = 50	0.00643056	7.90E-07
	m = 60	0.00643056	6.41E-07
	m = 70	0.00643056	5.40E-07
	m = 80	0.00643056	4.66E-07
	m = 90	0.00643056	4.37E-07
	m = 100	0.00643056	4.11E-07

*Relative errors are calculated based on the values of probabilities to 16 decimal places

M=1000

parameter	method	probability	relative error
λ=2、α=2	exact	0.0137559	―
	previous study	0.0137673	0.000828
	approximation		
	m = 10	0.0137560	7.49E-06
	m = 20	0.0137559	1.44E-09
	m = 30	0.0137559	2.40E-13
	m = 40	0.0137559	7.31E-15
	m = 50	0.0137559	0
	m = 60	0.0137559	0
	m = 70	0.0137559	0
	m = 80	0.0137559	0
	m = 90	0.0137559	0
	m = 100	0.0137559	0
λ=4、α=4	exact	0.0114389	―
	previous study	0.0114413	0.000213
	approximation		
	m = 10	0.0114389	2.11E-09
	m = 20	0.0114389	0
	m = 30	0.0114389	0
	m = 40	0.0114389	0
	m = 50	0.0114389	0
	m = 60	0.0114389	0
	m = 70	0.0114389	0
	m = 80	0.0114389	8.80E-15
	m = 90	0.0114389	8.80E-15
	m = 100	0.0114389	8.80E-15
λ=2、α=3	exact	0.0150798	―
	previous study	0.0150919	0.000803
	approximation		
	m = 10	0.0150797	3.11E-06
	m = 20	0.0150797	1.94E-06
	m = 30	0.0150798	1.35E-06
	m = 40	0.0150798	1.03E-06
	m = 50	0.0150798	8.38E-07
	m = 60	0.0150798	7.06E-07
	m = 70	0.0150798	6.10E-07
	m = 80	0.0150798	5.38E-07
	m = 90	0.0150798	4.81E-07
	m = 100	0.0150798	4.35E-07
λ=3、α=5	exact	0.0128245	―
	previous study	0.0128293	0.000369
	approximation		
	m = 10	0.0128245	1.36E-06
	m = 20	0.0128245	7.42E-07
	m = 30	0.0128245	5.13E-07
	m = 40	0.0128245	3.93E-07
	m = 50	0.0128245	3.19E-07
	m = 60	0.0128245	2.69E-07
	m = 70	0.0128245	2.32E-07
	m = 80	0.0128245	2.05E-07
	m = 90	0.0128245	1.83E-07
	m = 100	0.0128245	1.65E-07

*Relative errors are calculated based on the values of probabilities to 16 decimal places

M=1500

parameter	method	probability	relative error
λ=2、α=2	exact	0.0205684	—
	previous study	0.0205797	0.000549
	approximation		
	m = 10	0.0205686	7.91E-06
	m = 20	0.0205684	1.72E-09
	m = 30	0.0205684	4.57E-13
	m = 40	0.0205684	4.89E-15
	m = 50	0.0205684	4.89E-15
	m = 60	0.0205684	4.89E-15
	m = 70	0.0205684	4.89E-15
	m = 80	0.0205684	4.89E-15
	m = 90	0.0205684	4.89E-15
	m = 100	0.0205684	4.89E-15
λ=4、α=4	exact	0.0171104	—
	previous study	0.0171128	0.000140
	approximation		
	m = 10	0.0171104	2.22E-09
	m = 20	0.0171104	0
	m = 30	0.0171104	0
	m = 40	0.0171104	0
	m = 50	0.0171104	0
	m = 60	0.0171104	5.88E-15
	m = 70	0.0171104	0
	m = 80	0.0171104	5.88E-15
	m = 90	0.0171104	5.88E-15
	m = 100	0.0171104	5.88E-15
λ=2、α=3	exact	0.0225402	—
	previous study	0.0225522	0.000533
	execution time		
	m = 10	0.0225401	1.90E-06
	m = 20	0.0225401	1.29E-06
	m = 30	0.0225401	8.93E-07
	m = 40	0.0225402	6.85E-07
	m = 50	0.0225402	5.56E-07
	m = 60	0.0225402	4.69E-07
	m = 70	0.0225402	4.05E-07
	m = 80	0.0225402	3.57E-07
	m = 90	0.0225402	3.19E-07
	m = 100	0.0225402	2.88E-07
λ=3、α=5	exact	0.0191774	—
	previous study	0.0191820	0.000244
	approximation		
	m = 10	0.0191773	9.01E-07
	m = 20	0.0191773	4.93E-07
	m = 30	0.0191774	3.41E-07
	m = 40	0.0191774	2.61E-07
	m = 50	0.0191774	2.12E-07
	m = 60	0.0191774	1.79E-07
	m = 70	0.0191774	1.54E-07
	m = 80	0.0191774	1.36E-07
	m = 90	0.0191774	1.22E-07
	m = 100	0.0191774	1.10E-07

*Relative errors are calculated based on the values of probabilities to 16 decimal places
