# Supplementary material for: A numerical approach for a discrete Markov model for progressing drug resistance of cancer
Source: PLoS Comput Biol. 2019 Feb 19;15(2):e1006770. doi: 10.1371/journal.pcbi.1006770 (PMC6396936; doi:10.1371/journal.pcbi.1006770)
Supplement: S2 Table — (PDF) [file pcbi.1006770.s003.pdf]

$\lambda=2$、$\alpha=2$

Size	10000	30000	50000	100000	1000000
Median	0.129486	0.340348	0.499996	0.749916	0.999999
95% Confidential interval(lower)	0.129251	0.339994	0.499623	0.749652	0.999998
95% Confidential interval(upper)	0.129720	0.340702	0.500370	0.750180	1.000000
approximation (A)	0.129440	0.340239	0.499995	0.749998	0.999999
previous study (B)	0.129449	0.340246	0.500000	0.750000	0.999999
Δ(B-A)	0.000009	0.000007	0.000005	0.000002	0.000000

$\lambda=4$、$\alpha=4$

Size	10000	30000	50000	100000	1000000
Median	0.108801	0.292055	0.437466	0.683529	0.999988
95% Confidential interval(lower)	0.108522	0.291844	0.437171	0.683134	0.999985
95% Confidential interval(upper)	0.109081	0.292266	0.437761	0.683923	0.999991
approximation (A)	0.108697	0.291934	0.437500	0.683594	0.999990
previous study (B)	0.108699	0.291934	0.437500	0.683594	0.999990
Δ(B-A)	0.000002	0.000000	0.000000	0.000000	0.000000

$\lambda=2$、$\alpha=3$

Size	10000	30000	50000	100000	1000000
Median	0.141098	0.366375	0.532464	0.781514	1.000000
95% Confidential interval(lower)	0.140808	0.366086	0.532256	0.781204	1.000000
95% Confidential interval(upper)	0.141388	0.366664	0.532671	0.781823	1.000000
approximation (A)	0.141060	0.366310	0.532489	0.781437	1.000000
previous study (B)	0.141071	0.366318	0.532495	0.781439	1.000000
Δ(B-A)	0.000011	0.000008	0.000006	0.000002	0.000000

$\lambda=3$、$\alpha=5$

Size	10000	30000	50000	100000	1000000
Median	0.121147	0.321327	0.475857	0.725097	0.999997
95% Confidential interval(lower)	0.120872	0.321125	0.475607	0.724718	0.999995
95% Confidential interval(upper)	0.121422	0.321529	0.476108	0.725476	1.000000
approximation (A)	0.121130	0.321156	0.475658	0.725066	0.999998
previous study (B)	0.121134	0.321159	0.475659	0.725067	0.999998
Δ(B-A)	0.000004	0.000003	0.000001	0.000001	0.000000
