# Supplementary material for: A numerical approach for a discrete Markov model for progressing drug resistance of cancer
Source: PLoS Comput Biol. 2019 Feb 19;15(2):e1006770. doi: 10.1371/journal.pcbi.1006770 (PMC6396936; doi:10.1371/journal.pcbi.1006770)
Supplement: S3 Table — (PDF) [file pcbi.1006770.s004.pdf]

	Size	10000	30000	50000	100000	1000000
approximation	λ =2、 α =2	170.270734	514.855166	869.318045	1763.206138	42275.633
	λ =4、 α =4	173.303914	536.151129	904.469668	1831.573315	52959.828
	λ =2、 α =3	170.193927	514.472084	863.701761	1757.699664	40835.519
	λ =3、 α =5	173.260736	524.346106	885.748747	1803.101197	46316.275
simulation	λ =2、 α =2	10733.56372	32223.22758	53739.57229	108444.1208	1100468.239
	λ =4、 α =4	5519.786092	16550.76083	27679.97225	56226.76364	563124.7137
	λ =2、 α =3	5519.786092	16550.76083	27679.97225	56226.76364	1101224.975
	λ =3、 α =5	6907.72127	20363.08368	33852.14323	68402.14659	704800.6809
